# Correction to “Intratumoral *Fusobacterium nucleatum* Drives Cancer‐Associated Fibroblasts Enrichment and Immune Exclusion in Esophageal Squamous Cell Carcinoma”

**DOI:** 10.1002/ags3.70253

**Published:** 2026-07-21

T. Ofuchi , K. Shinjo , Q. Hu , et al., “Intratumoral *Fusobacterium nucleatum* Drives Cancer‐Associated Fibroblasts Enrichment and Immune Exclusion in Esophageal Squamous Cell Carcinoma,” Annals of Gastroenterological Surgery 10, no. 2 (2025): 611–620. 10.1002/ags3.70116 PMC1296202541799583

The author list for the above article was incorrect. Dr. Yutaka Kondo and Dr. Keiko Shinjo should be added as co‐authors. The final author list is as follows:

Takashi Ofuchi^1,2^, Keiko Shinjo^3^, Qingjiang Hu^1^, Kazuki Omachi^1,2^, Kosuke Kanemitsu^2^, Hajime Otsu^1^, Yusuke Yonemura^1^, Taro Tobo^4^, Yoshifumi Baba^5^, Masaaki Iwatsuki^2^, Yutaka Kondo^3^, and Koshi Mimori^1^.

^1^Department of Surgery Beppu Hospital, Kyushu University, Oita Japan

^2^Department of Gastroenterological Surgery Graduate School of Medical Sciences, Kumamoto University, Kumamoto Japan

^3^Division of Cancer Biology, Nagoya University Graduate School of Medicine, 65 Tsurumai‐cho, Showa‐ku, Nagoya, Japan

^4^Department of Pathology Beppu Hospital, Kyushu University Oita Japan

^5^Department of Gastrointestinal Surgery Graduate School of Medicine, The University of Tokyo, Tokyo Japan

The online article has been corrected.

We apologize for this error.

